# Research skills of Queensland Health podiatrists: how do they rate and are they improving?

**DOI:** 10.1186/1757-1146-6-S1-P9

**Published:** 2013-05-31

**Authors:** Peter A Lazzarini, Julia Geraghty, Ewan M Kinnear, Mark Butterworth, Donna Ward

**Affiliations:** 1 Allied Health Research Collaborative, Metro North Hospital & Health Service, Queensland Health, Brisbane, Queensland, 4032, Australia; 2 Department of Podiatry, Metro North Hospital & Health Service, Queensland Health, Brisbane, Queensland, 4032, Australia; 3 School of Clinical Sciences, Queensland University of Technology, Brisbane, Queensland, 4059, Australia; 4 Department of Allied Health Services, Metro North Hospital & Health Service, Queensland Health, Brisbane, Queensland, 4032, Australia; 5 Department of Clinical Psychology & Neuropsychology, The Prince Charles Hospital, Queensland Health, Brisbane, Queensland, 4032, Australia

## Background

For health professions to evolve in a highly evidence-based world it seems imperative for clinicians to build their research skills. This observational paper aims to report the research skill levels of statewide public-sector podiatrists at two different time points twelve-months apart.

## Methods

The Research Capacity & Culture (RCC) survey was distributed to all Queensland Health podiatrists in January 2011 (n=58) and January 2012 (n=60). The RCC is a validated tool designed to measure indicators of research skill in health professionals. Participants rate skill levels against each individual, team and organisation statement on a 10-point scale (1=lowest, 10=highest). Chi-squared and Mann Whitney U tests were used to determine any differences between survey samples.

## Results

Thirty-seven (64%) podiatrists responded to the 2011 survey and 33 (55%) the 2012 survey. The 2011 survey respondents reported low skill levels (Median<4) on most individual research aspects. However, most reported their organisations’ skills to perform and support research at higher levels (Median>6). The 2012 survey respondents reported significantly higher skill levels compared to the 2011 survey in individuals’ ability to secure research funding, submit ethics applications and provide research advice (p<0.05).

## Conclusions

This study reports the research skill levels of the largest podiatry populations to date. The 2011 findings indicated that podiatrists have similarly low research skill levels to those reported in the generic allied health literature. The 2012 findings suggest podiatrists perceived higher skills and support to initiate research than in 2011. This improvement coincided with podiatry research capacity building strategies.

